# Exploring Aging Masculinities From Different Disciplines and Perspectives

**DOI:** 10.1093/geroni/igaa057.3014

**Published:** 2020-12-16

**Authors:** Liat Ayalon, Josep Armengol, Michael Kimmel

## Abstract

Traditionally, gerontology research has been relatively genderless. When the intersection of age and gender was explored, this was done primarily by focusing on the experiences of older women. Much less is known about the experiences of older men. The present symposium brings together work from the humanities and the social sciences in order to explore societal images and personal experiences of aging men. The paper by Maierhofer and Ratzenböck provides a theoretical outlook on this intersection from the humanities perspective, followed by empirical applications from the social sciences. Next, Armengol uses contemporary American literature to challenge the traditional stereotype of decline in sexuality and masculinity. The paper by Ni Leime & O’Neill examines stereotypes of aging masculinities, but this time from the perspective of older men as the audience who react to their portrayal in visual culture. Finally, Ayalon and Gweyrtz-Meydan present ethical dilemmas faced by physicians who treat older men’s sexuality in light of active marketing campaigns of the pharmaceutical industry, which advocate for a model of successful aging and ongoing sexual intercourse. The discussant, Kimmel, will conceptualize the four papers by stressing the different types of information that can be obtained via different methods of inquiry. The complementary information provided by the different papers and the integration of methods and findings from the humanities with the social sciences will be discussed.

